# The *Sox2* promoter-driven CD63-GFP transgenic rat model allows tracking of neural stem cell-derived extracellular vesicles

**DOI:** 10.1242/dmm.028779

**Published:** 2018-01-01

**Authors:** Aya Yoshimura, Naoki Adachi, Hitomi Matsuno, Masaki Kawamata, Yusuke Yoshioka, Hisae Kikuchi, Haruki Odaka, Tadahiro Numakawa, Hiroshi Kunugi, Takahiro Ochiya, Yoshitaka Tamai

**Affiliations:** 1 Division of Laboratory Animals Resources, National Institute of Neuroscience, National Center of Neurology and Psychiatry (NCNP), 4-1-1 Ogawa-Higashi, Kodaira, Tokyo 187-8502, Japan; 2 Department of Mental Disorder Research, National Institute of Neuroscience, NCNP, 4-1-1 Ogawa-Higashi, Kodaira, Tokyo 187-8502, Japan; 3 Education and Research Facility of Animal Models for Human Diseases, Center for Research Promotion and Support, Fujita Health University, 1-98 Dengakugakubo, Kutsukake-cho, Toyoake, Aichi 470-1192, Japan; 4 Department of Biomedical Chemistry, School of Science and Technology, Kwansei Gakuin University, 2-1 Gakuen, Sanda, Hyogo 669-1337, Japan; 5 Division of Molecular and Cellular Medicine, National Cancer Center Research Institute (NCC), 1-1 Tsukiji 5-chome, Chuo-ku, Tokyo 104-0045, Japan; 6 Department of Degenerative Neurological Diseases, National Institute of Neuroscience, NCNP, 4-1-1 Ogawa-Higashi, Kodaira, Tokyo 187-8502, Japan; 7 Department of Life Science and Medical Bioscience, School of Advanced Science and Engineering, Waseda University, 3-4-1 Okubo, Shinjuku-ku, Tokyo 169-8555, Japan; 8 Department of Cell Modulation, Institute of Molecular Embryology and Genetics, Kumamoto University, 2-2-1 Honjo, Chuo-ku, Kumamoto 860-0811, Japan; 9 Chromocenter Inc., 6-7-4 Minatojima-minami, Chuo-ku, Kobe, Hyogo 650-0047, Japan

**Keywords:** Model animals, Extracellular vesicles, CD63, Neural stem cells

## Abstract

Extracellular vesicles (EVs) can modulate microenvironments by transferring biomolecules, including RNAs and proteins derived from releasing cells, to target cells. To understand the molecular mechanisms maintaining the neural stem cell (NSC) niche through EVs, a new transgenic (Tg) rat strain that can release human CD63-GFP-expressing EVs from the NSCs was established. Human CD63-GFP expression was controlled under the rat *Sox2* promoter (Sox2/human CD63-GFP), and it was expressed in undifferentiated fetal brains. GFP signals were specifically observed in *in vitro* cultured NSCs obtained from embryonic brains of the Tg rats. We also demonstrated that embryonic NSC (eNSC)-derived EVs were labelled by human CD63-GFP. Furthermore, when we examined the transfer of EVs, eNSC-derived EVs were found to be incorporated into astrocytes and eNSCs, thus implying an EV-mediated communication between different cell types around NSCs. This new Sox2/human CD63-GFP Tg rat strain should provide resources to analyse the cell-to-cell communication via EVs in NSC microenvironments.

## Abstract (precis)

**Summary:** We generated a new transgenic rat model expressing CD63-GFP driven by the *Sox2* promoter, which enables the tracking and analysis of neural stem cell-derived extracellular vesicles.

## INTRODUCTION

Neural stem cells (NSCs) persist and generate new neurons in the adult mammalian brain in addition to the developing fetal brain. Neurogenesis during prenatal development is responsible for brain growth, whereas adult neurogenesis contributes to learning and memory throughout life (Deng et al., 2010). Furthermore, recent studies have suggested that decreased adult neurogenesis is involved in neuropsychiatric diseases, such as epilepsy and depression (Braun and Jessberger, 2014). To continue neuronal production, NSCs are present mainly in two regions, the subgranular zone (SGZ) of the hippocampus and the subventricular zone (SVZ) in the lateral wall of the lateral ventricles in the adult brain, and NSC proliferation and differentiation are regulated via extracellular stimulation from the stem cell niche (Riquelme et al., 2008). Therefore, research has focused on understanding the molecular mechanisms maintaining the NSC microenvironment.

Extracellular vesicles (EVs) are important microenvironmental factors (Fujita et al., 2015). Many different cell types in the body release EVs into the extracellular environment; they contain lipids, proteins, mRNAs and microRNAs (miRNAs) that originate from the releasing cells, and these biomolecules are transferred to target cells via the uptake of EVs. A number of cancer studies have reported that EVs mediate communication within the tumour microenvironment, contributing to cancer progression (Hu et al., 2015) and metastasis (Peinado et al., 2012; Tominaga et al., 2015). In the central nervous system (CNS), neurons, astrocytes, oligodendrocytes and microglia have been reported to secrete EVs, which influence synaptic plasticity and the immune system. For example, neurons release EVs carrying the GluR2/3 subunits of AMPA receptors depending on enhanced glutamatergic activity (Lachenal et al., 2011), and astrocytes shed EVs carrying Hsp70 in response to heat stress to protect neurons (Taylor et al., 2007). Moreover, EVs have been shown to remove obsolete proteins from cells (Danzer et al., 2012; Rajendran et al., 2006). Although these studies have suggested that EVs function to maintain CNS homeostasis, whether EVs have influences on shaping and maintaining the niche around NSCs and how EVs move between cells in the NSC region remain unclear. Generally, most *in vitro* and *in vivo* assays so far use EVs isolated from cultured donor cells, and imaging is performed using cell membrane-tracking reagents, such as PKH dyes, or transfection of fluorescent-tagged EV marker proteins to examine the transfer of EV contents into the recipient cells (Grapp et al., 2013; Koumangoye et al., 2011; Suetsugu et al., 2013). However, the behaviour of EVs in cultured cells might not reflect that of EVs released *in vivo*. To address this challenge, we generated a new transgenic (Tg) rat model expressing fluorescent-tagged EVs in the NSC region.

EVs are classified into diverse types depending on their membrane origin, either the endosomal or the plasma membrane, and are called exosomes or microvesicles, respectively (Raposo and Stoorvogel, 2013). Exosomes are vesicles, around 40−100 nm in diameter, produced from late endosomes/multivesicular bodies (MVBs) formed by invagination and budding of early endosomes, whereas microvesicles are larger EVs (up to ∼1000 nm in diameter) generated by outward budding of the plasma membrane. The CD63 [also known as lysosomal-associated membrane protein 3 (LAMP3)] is an EV marker, in particular an exosome marker, because it is highly enriched in late endosomes via an intracellular pathway from the trans-Golgi network or via endocytosis from the cell surface (Pols and Klumperman, 2009). A fluorescent-tagged *CD63* gene has been transfected into cultured cell lines, and *in vivo* in *Drosophila melanogaster*, to detect EV release from donor cells and EV transfer into recipient cells (Corrigan et al., 2014; Gross et al., 2012; Koumangoye et al., 2011; Suetsugu et al., 2013; Sung et al., 2015). In addition, we previously generated a Tg rat strain expressing human CD63-copGFP under the control of the ubiquitous CAG promoter (CAG/human CD63-GFP) and showing human CD63-GFP labelled EVs in body fluids (Yoshimura et al., 2016b). In the present study, we constructed a human CD63-copGFP gene regulated by the *Sox2* promoter (Sox2/human CD63-GFP). The transcription factor gene *Sox2* is expressed in the NSCs of both embryonic and adult brains and is required for the maintenance of NSCs (Ferri et al., 2004). Therefore, it is supposed that Sox2/human CD63-GFP rats have GFP-labelled EVs in NSCs.

Here, we demonstrated that exogenous human CD63-GFP expression was detected in the NSCs of the Tg rats and that the human CD63-GFP labels were detected in embryonic NSC (eNSC)-derived EVs in recipient cells *in vitro*.

## RESULTS AND DISCUSSION

### Sox2/human CD63-GFP Tg rats express GFP in diverse tissues, including the developing brain

**Fig. 1.**
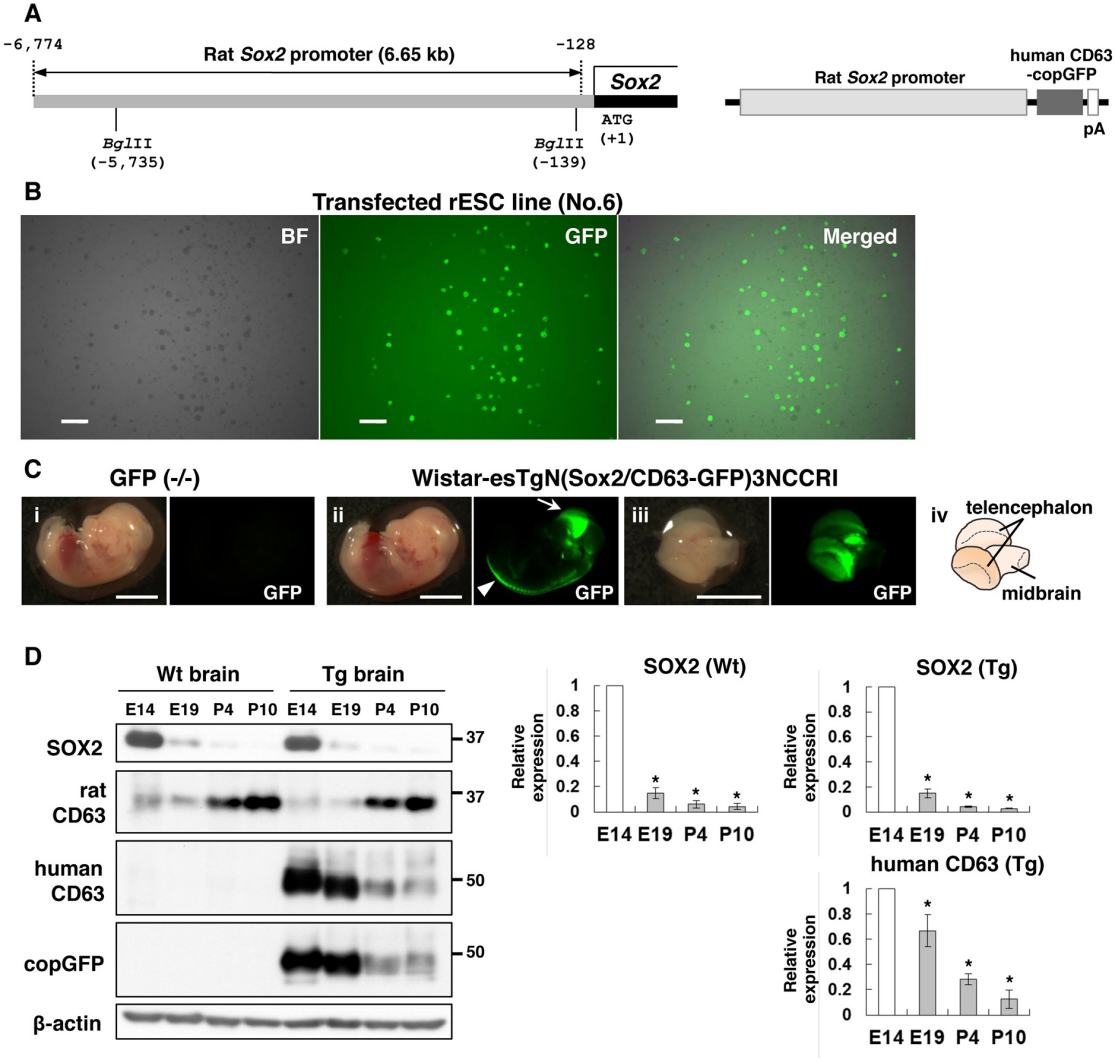
**Generation of Tg rats by transfection of Sox2/human CD63-GFP gene into rESCs.** (A) The localization of *Sox2* promoter including two *Bgl*II sites (left) and structure of the transgene construction (right). (B) Photomicrograph of No. 6 rESC line transfected with the Sox2/human CD63-GFP gene. The rESCs expressed GFP ubiquitously. Scale bars: 300 µm. (C) E14 rats obtained by mating a Tg [Wistar-esTgN(Sox2/CD63-GFP)3NCCRI] male rat with a Wt female rat. The fetus not expressing GFP is a non-Tg littermate (i). The Tg fetus showed a high level of GFP fluorescence in the spinal cord (ii, arrowhead) and telencephalon (ii, arrow, iii and iv). Scale bars: 5 mm. (D) Western blot analysis for SOX2, endogenous rat CD63, exogenous human CD63 and copGFP during rat brain development. The lysates were collected from the telencephalon at E14 and the cerebral cortex at E19, P4 and P10 (*n*=3). β-actin was used as a loading control. Error bars represent the s.d. (*P<0.001 versus E14, one-way ANOVA with Bonferroni's post hoc test).

To investigate communication via EVs in specific regions of the brain, we generated a Sox2/human CD63-GFP Tg rat strain using rat embryonic stem cells (rESCs). The vector encoding human CD63-GFP under control of the proximal 6.65 kb rat *Sox2* promoter was transfected into rESCs (Fig. 1A); several rESC colonies showed GFP fluorescence because SOX2 is essential for maintaining self-renewal in ESCs. Three rESC lines (No. 6, No. 10 and No. 22) indicated a bright and stable fluorescence at the passages shown in Fig. 1B and Fig. S1; therefore, these lines were used to produce chimaeras. Two rESC lines (No. 6 and No. 22) and nine rats (two males and seven females) showed coat colour chimaerism resulting from the injection of GFP rESCs into blastocysts. As the rESC line used in this study was established from female blastocysts of Wistar rats (Kawamata and Ochiya, 2010), the seven chimaeric females were bred to Wistar males. One female chimaeric rat originating from No. 6 rESC line produced a GFP-positive male, thus indicating the successful germline transmission of the transgene, and it was named the Wistar-esTgN(Sox2/CD63-GFP)3NCCRI strain.

The GFP fluorescence was observed in the telencephalon and along the spinal cord of the embryonic day (E)14 fetal rats (Fig. 1C), indicating that the *Sox2* promoter fragment contains regulatory elements for region-specific expression. Similar expression patterns were observed in previous studies using the regulatory elements of the *Sox2* promoter in mice (Kang and Hébert, 2012; Zappone et al., 2000). SOX2 expression was downregulated in the developing cerebral cortex (Fig. 1D). Consistent with its expression pattern, exogenous human CD63 and copGFP exhibited reduced expression in the cerebral cortex of postnatal rats (Fig. 1D).

**Fig. 2.**
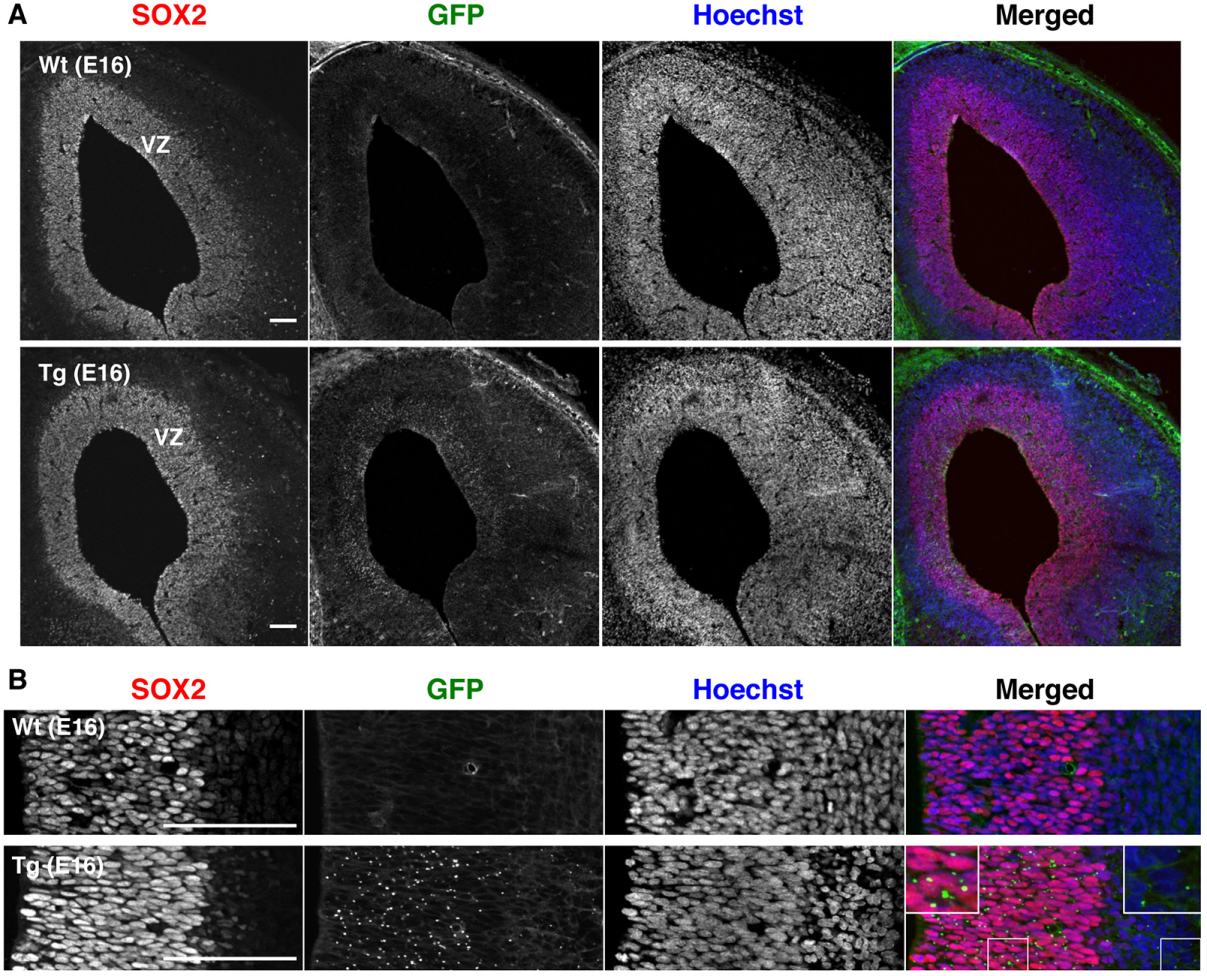
**Immunohistological analysis of the telencephalon of Tg rat embryo.** (A,B) Low (A) and high (B) magnification of coronal sections of the telencephalon demonstrating immunoreactivity of SOX2 and GFP fluorescence. (B) Tg rat telencephalon images showed GFP signals (green) not only around SOX2-positive cells (red) of the VZ but also in SOX2-negative cells (blue), as shown in the magnified view (white boxes). No GFP signals was observed in the Wt telencephalon. Scale bars: 100 µm.

By contrast, the expression of endogenous rat CD63 was increased depending on the development of the cerebral cortex. In the developing telencephalon at E16, immunohistological analysis showed SOX2 expression along the ventricular zone (VZ) (Fig. 2A). A punctate distribution of GFP was observed in the SOX2-positive region of the Tg telencephalon, but not in the wild type (Wt) (Fig. 2B; Fig. S2). The GFP signals were also observed in SOX2-negative region, implying the possibility of EV transfer in physiological conditions. In the adult brain of Tg rats, the GFP fluorescent signals were also detected in some SOX2-positive cells in the SVZ (Fig. S3, arrows). Unexpectedly, intense GFP signals were distributed along the blood vessels, indicated by lectin immunoreactivity (Fig. S3). These GFP signals seemed to be localized at the feet of astrocytes contacting the blood vessel (Fig. S3, arrowheads). These images indicate that endothelial cells and/or pericytes that form the blood-brain barrier with astrocytes contain human CD63-GFP. Considering that serum EVs do not carry detectable levels of human CD63-GFP (see Fig. 4B), GFP signals around the blood vessels are probably attributable to human CD63-GFP expressed in these cells or to GFP-labelled EVs taken up and accumulated from other SOX2-expressing cells, although further detailed studies are required. Furthermore, the adult hippocampus of Tg rats showed a normal distribution of NeuN (neuronal nuclei)-positive neurons (Fig. S4).

**Fig. 3.**
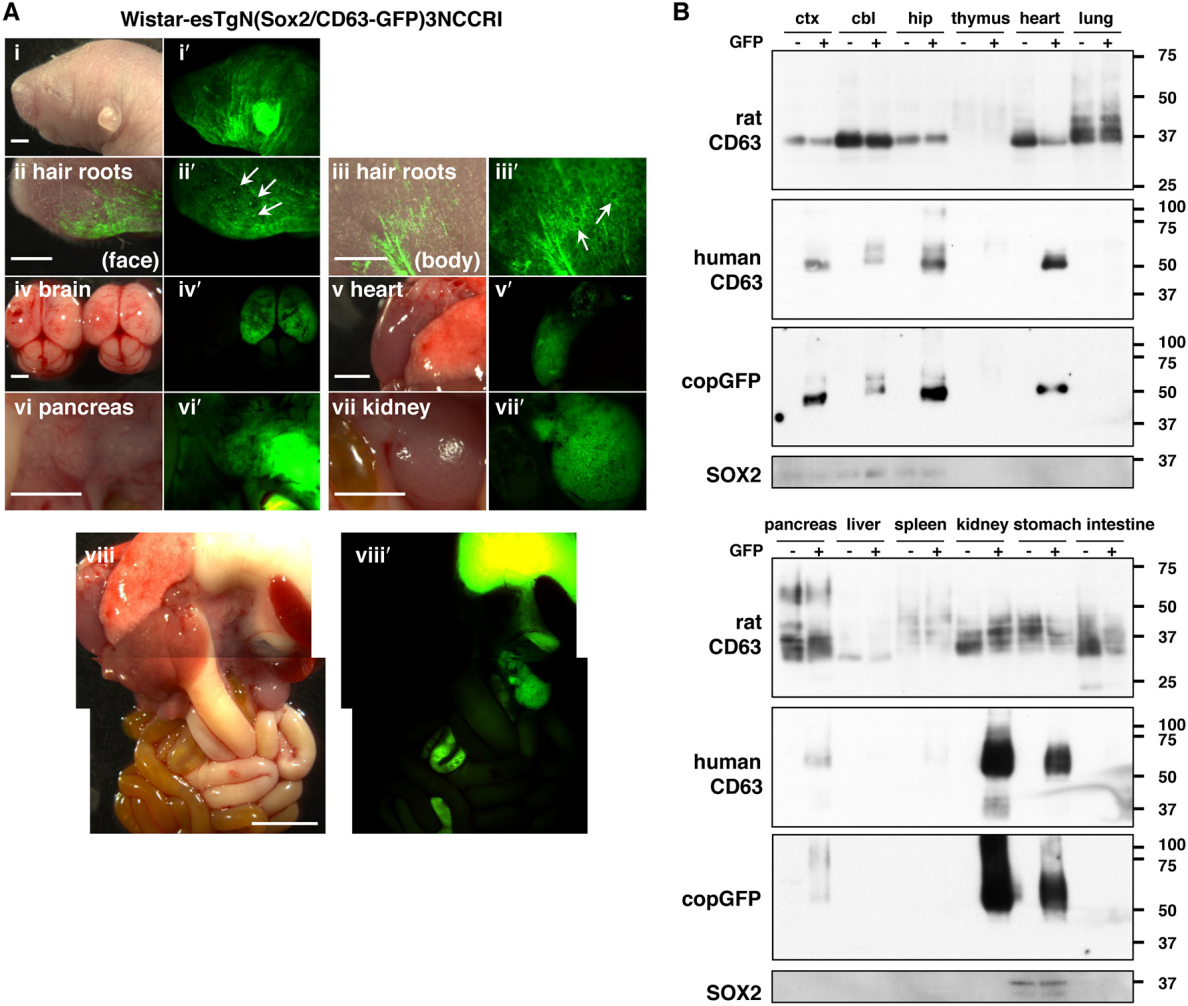
**Human CD63-GFP expression in the major organs of neonatal Wistar-esTgN(Sox2/CD63-GFP)3NCCRI Tg rats.** (A) Pictures from Tg offspring at P2 (ii and iii, merged; i′-viii′, GFP). The hair roots in the whiskers (ii and ii′, arrows), but not in the body (iii and iii′, arrows), expressed GFP fluorescence. The skin, brain, heart, kidney and stomach showed strong fluorescent signals, and pancreas showed a weaker fluorescence. Scale bars: 2 mm (i, ii, iv-vii), 1 mm (iii) and 5 mm (viii). (B) Expression of endogenous rat CD63, exogenous human CD63, copGFP and SOX2 in the tissue lysates from GFP-negative (−) and GFP-positive (+) offspring at P4. cbl, cerebellum; ctx, cortex; hip, hippocampus.

Human CD63-GFP expression was detected in other neonatal tissues in addition to the brain (Fig. 3A,B). A high level of GFP fluorescence was observed in the skin and heart, and especially in the kidney and stomach (Fig. 3Aviii′). Furthermore, hair follicles in the whiskers (Fig. 3Aii′, arrows) but not on the body (Fig. 3Aiii′, arrows) expressed GFP fluorescence, whereas the pancreas showed a weaker fluorescence. Slight signals were detected in the thymus and spleen by western blot analysis with anti-human CD63 and copGFP antibody, whereas GFP fluorescence was not observed in these tissues. SOX2 signals in western blotting were detected in brain tissues and the stomach, but not in the kidney, in which very high levels of GFP fluorescence and human CD63 expression were present (Fig. 3A,B). Previous studies also reported expression of *Sox2* in the stomach and other tissues in mice and humans (Arnold et al., 2011; Cimpean et al., 2011; Driskell et al., 2009; Kang and Hébert, 2012; Raghoebir et al., 2012; Riekstina et al., 2009). However, they did not mention the expression of *Sox2* in the kidney. Considering that a high level of human *CD63* mRNA expression was also observed in the kidney of the Tg rat (Fig. S5), it is likely that the human CD63-GFP protein originated from the kidney itself, not from EVs derived from other tissues and accumulated in the kidney. Therefore, human CD63-GFP was expressed in the kidney of our Tg rats because the kidney-specific regulatory element would be present in the 6.65 kb fragment or in the inserted chromosomal region of the Sox2/human CD63-GFP vector.

We previously generated another Tg rat strain (CAG/human CD63-GFP), resulting in expression throughout the body (Yoshimura et al., 2016b). Although the CAG/human CD63-GFP Tg rats showed embryonic lethality in males and premature death in females, the Sox2/human CD63-GFP Tg rats in the present study showed no lethality or infertility in either sex. However, the Sox2/human CD63-GFP Tg rats exhibited frequent water drinking behaviour and a high urine output, with no significant difference between male and female individuals. A previous report by another group found that CD63-deficient mice displayed an increased urine output (Schröder et al., 2009), and it was also shown that the transfection of CD63-FLAG in Cos7 cells led to internalization of endocytosed gastric-type H,K-ATPase β-subunit (HKβ) into CD63-positive vesicles to prevent the recycling of HKβ to the cell surface (Duffield et al., 2003). These results suggested that CD63 in the kidney might strongly affect the functions of other membrane proteins, such as potassium channels, that are involved in epithelial electrolyte and water transport. Importantly, CAG/human CD63-GFP Tg rats in a previous study did not demonstrate this phenomenon even though the kidney expressed very high levels of human CD63-GFP (Yoshimura et al., 2016b). This is probably attributable to the different human CD63-GFP expression patterns depending on the cell type, via the control of different promoters.

### Characterization of EVs isolated from serum of human CD63-GFP Tg rats

**Fig. 4.**
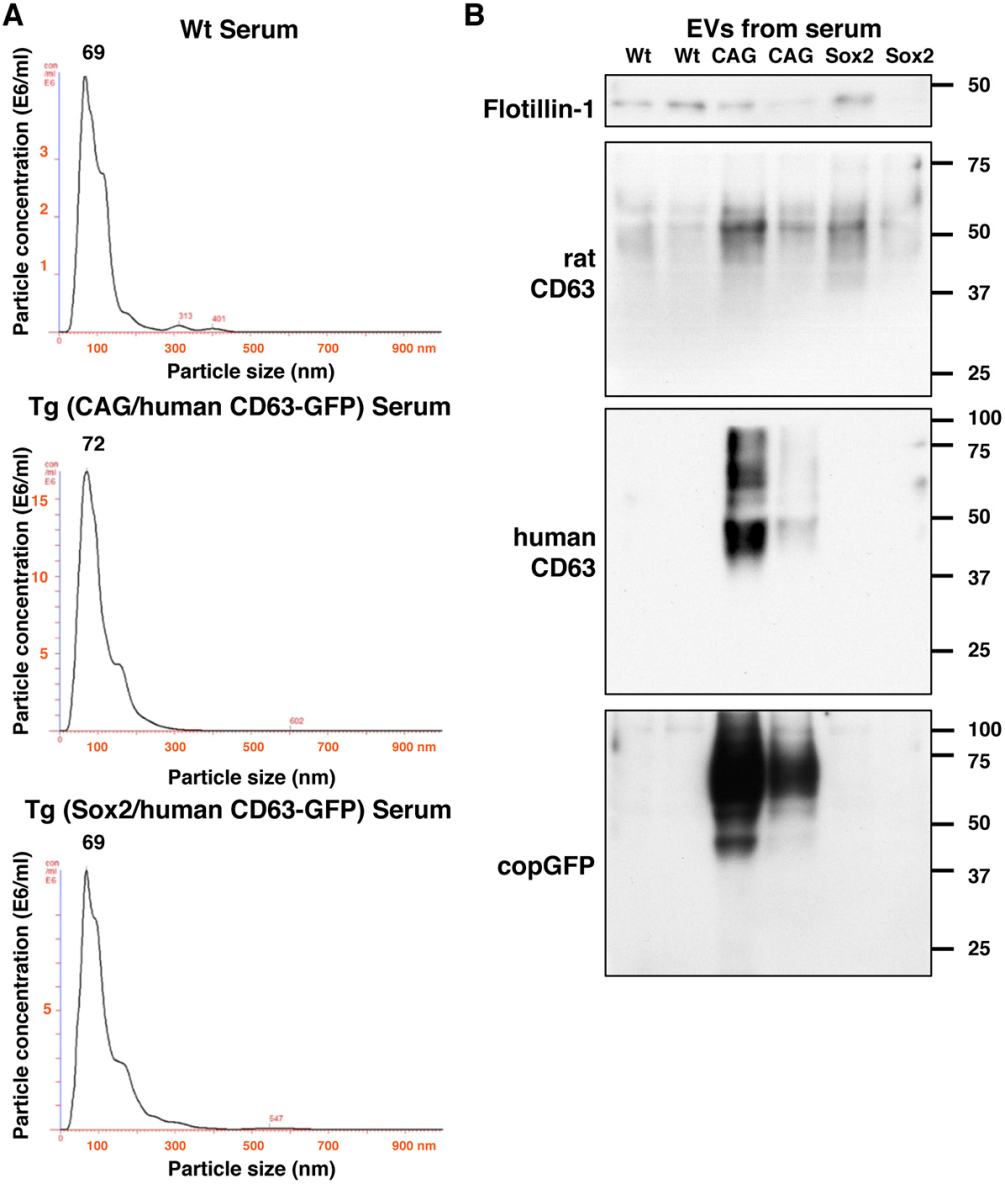
**Identification and characterization of the EVs isolated from the serum of Wt and two Tg rats [Wistar-esTgN(Sox2/CD63-GFP)3NCCRI and Wistar-esTgN(CAG/CD63-GFP)3NCCRI].** (A) Size distribution of the EVs collected by ultracentrifugation was determined using the NanoSight system. (B) Western blot analysis of the EVs for flotillin-1, rat CD63, human CD63 and copGFP (*n*=2).

The Tg rats in the present study showed specific human CD63-GFP transgene expression in tissues/cells driven by the *Sox2* promoter. It is essential to identify the releasing cells of EVs circulating in the body to reveal the transfer pathway in the body. To examine GFP-labelled EVs that were released into blood, we collected EVs from the serum of Wt, Sox2/human CD63-GFP and CAG/human CD63-GFP Tg rats using ultracentrifugation. NanoSight analysis showed that vesicles measuring around 50–100 nm were present in the serum of all three rat genotypes (Fig. 4A). To assess expression of the EV markers and exogenous human CD63-GFP, isolated pellets were examined by western blot analysis (Fig. 4B). All samples showed the presence of the EV markers rat CD63 and flotillin-1. The EV pellets from CAG/human CD63-GFP Tg rats were further characterized by the presence of human CD63 and copGFP, whereas these exogenous proteins were not detected in the EVs from Wt and Sox2/human CD63-GFP Tg rats. This is explained by region-restricted expression of the transgene in Sox2/human CD63-GFP Tg rats compared with CAG/human CD63-GFP Tg rats (Yoshimura et al., 2016b) or, alternatively, the tissues/cells expressing Sox2/human CD63-GFP might not be the principal donor cells of EVs circulating in the blood, because haematopoietic tissues in the transgenic rats, especially bone-marrow cells (Fig. S6), expressed smaller amounts of human CD63 and copGFP than those of CAG/human CD63-GFP Tg rats.

### Expression of Sox2/CD63-GFP in differentiated eNSCs

**Fig. 5.**
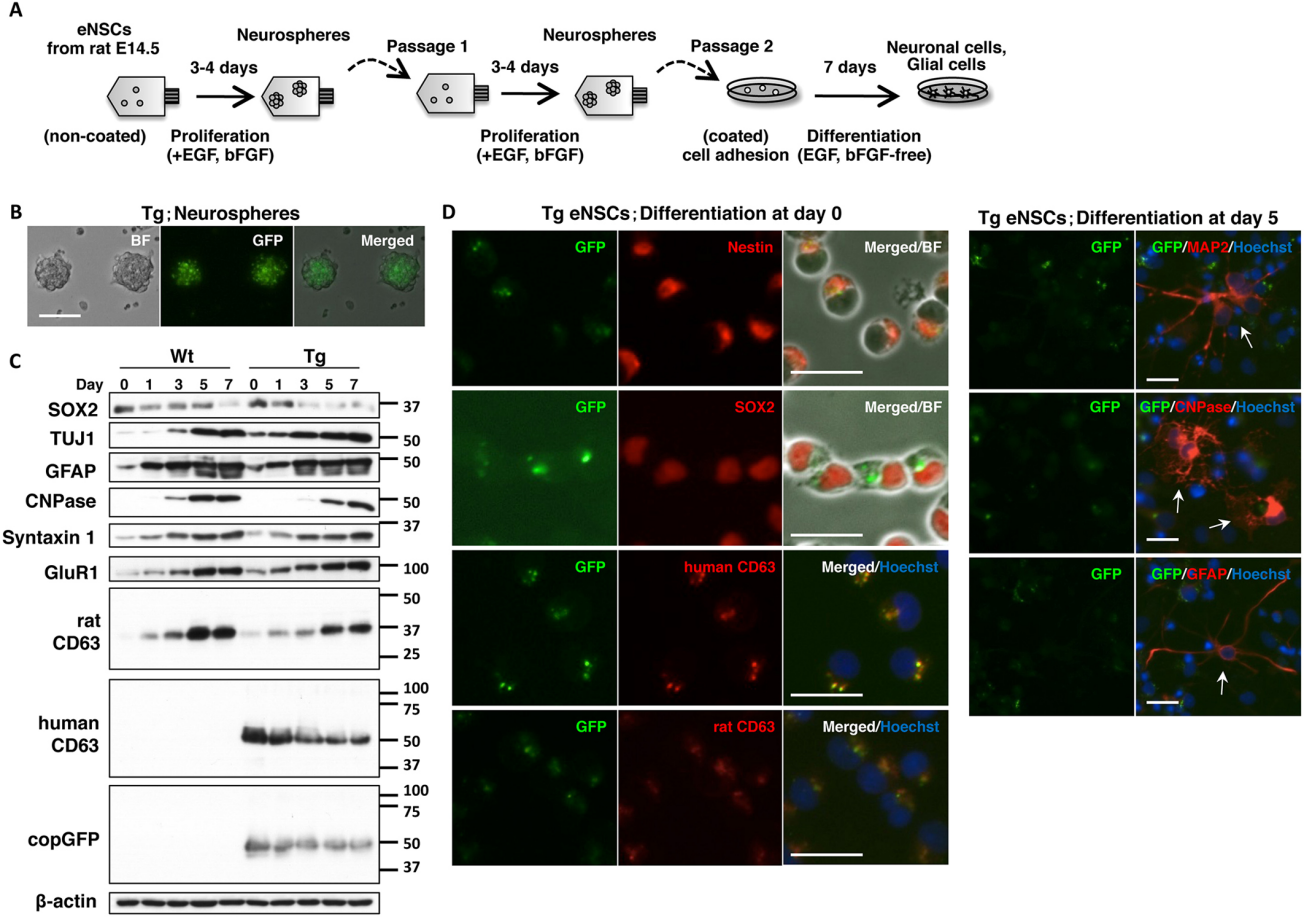
**Human CD63-GFP expression and localization during differentiation of eNSCs obtained from the telencephalon of Tg rats at E14.** (A) Schematic representation of the protocol for eNSC proliferation and differentiation *in vitro*. (B) GFP-positive neurospheres from Tg rats. Scale bar: 50 µm. (C) Protein expression profiles during differentiation of eNSCs. Markers for neurons (TUJ1), astrocytes (GFAP), oligodendrocytes (CNPase), neural stem cells (SOX2) and presynaptic (syntaxin 1) and postsynaptic (GluR1) proteins, rat CD63 and human CD63-GFP were examined in Wt and Tg rat cells. The experiment was performed once. (D) GFP expression in the cultured Tg rat cells at day 0 (left) and 5 days after differentiation (right). Immunostaining indicated the co-localization of GFP with human CD63-positive and with rat CD63-positive signals around nuclei (blue) in the nestin- and SOX2-positive undifferentiated cells at day 0. At day 5, no GFP signal was seen in the MAP2-positive, CNPase-positive and GFAP-positive differentiated cells (arrows). All the experiments except (C) were repeated at least two times using different cultures. Scale bars: 20 µm.

To investigate the expression of human CD63-GFP driven by the *Sox2* promoter in eNSCs and differentiated neural and glial cells, we used an *in vitro* model for eNSC differentiation (Fig. 5A). Rat eNSCs obtained from the telencephalon at E14 formed neurospheres in the presence of epidermal growth factor (EGF) and basic fibroblast growth factor (bFGF). Dissociated neurospheres were passaged twice, and cells were then induced to differentiate on pre-coated tissue culture dishes in EGF- and bFGF-free medium. After 7 days of differentiation, eNSCs developed a neuronal morphology with long and branched neurites or radial glial morphology. In the Tg neurospheres, all spheres expressed GFP (Fig. 5B; Fig. S7). Western blot analysis confirmed upregulation of neuron-specific protein (TUJ1), synaptic proteins (syntaxin 1 and GluR1), astrocyte-specific protein (GFAP) and oligodendrocyte-specific protein (CNPase) during *in vitro* differentiation of Tg eNSCs, similar to Wt eNSCs (Fig. 5C). Decreased expression of the NSC marker SOX2 was observed after differentiation, and the expression of human CD63 and copGFP in Tg samples was reduced in accord with the SOX2 expression. The expression of endogenous rat CD63 was increased after differentiation with a similar expression pattern in the developing cerebral cortex. These results indicated that eNSCs of the rat model differentiated normally and that expression of the transgene was well regulated by the *Sox2* promoter. This is also supported by the normal distribution of the NeuN-positive cells in the hippocampus of the adult brain (Fig. S4).

We observed the expression of human CD63-GFP during differentiation of eNSCs using immunostaining (Fig. 5D, left panels; Fig. S8). GFP signals were detected close to nuclei in Tg cells that were positive for the stem cell markers nestin and SOX2 at day 0, when the cells were in an undifferentiated state. The GFP fluorescence was co-localized with human CD63 and endogenous rat CD63. Five days after differentiation, GFP signals were no longer detected in differentiated cells, including neural cells (MAP2), oligodendrocytes (CNPase) and astrocytes (GFAP) (Fig. 5D, right panels, arrows). These results clearly showed that human CD63-GFP was expressed specifically in undifferentiated NSCs, and the expression of GFP was reduced after differentiation. Wt eNSCs lost SOX2 expression during the *in vitro* differentiation process and began to express differentiation markers at day 5 (Fig. S9A,B), such as MAP2 (neuron: Fig. S9B, upper panel, arrows) and CNPase (immature and mature oligodendrocytes: Fig. S9B, lower panel, arrowheads and arrow, respectively). By contrast, GFAP-positive cells showed SOX2 expression (Fig. S9C, arrows). It is known that SOX2 plays a role in proliferating cells, including not only NSCs but also proliferating astrocytes in the developing cerebral cortex (Bani-Yaghoub et al., 2006). However, GFP was not visible in the differentiated astrocytes from the eNSCs of Tg rats at day 5 because they were thought to lack proliferation. To activate the proliferation of astrocytes, we maintained the differentiated cells in astrocyte medium for 6-10 days after differentiation. When GFAP-positive cells had dramatically proliferated, and expanded on the bottom of the dishes (Fig. S9D), GFP fluorescence was detected in GFAP-positive cells of Tg rats (Fig. S9D, arrows). These results suggested that the differentiated astrocytes resumed human CD63-GFP expression associated with proliferation.

Taken together, we demonstrated in *in vitro* culture that Sox2/human CD63-GFP Tg rats showed GFP signals specific to NSCs and proliferating astrocytes from embryonic brains. When EVs are secreted into the extracellular space for cytophysiological functions, such as cell movement (Sung et al., 2015) and intercellular contact with adjacent recipient cells (Cicero et al., 2015), secretion sites of EVs and MVB polarization in donor cells could be indicated by using GFP or other fluorescent-tagged CD63 markers. In *Drosophila* development, the accumulation of secreted EVs and MVBs containing Wnt proteins in the imaginal disc can also be shown by using a CD63-GFP marker, demonstrating that the EVs are transporters of Wnt proteins that act as morphogens (Gross et al., 2012). The Sox2/human CD63-GFP Tg rats will be useful to find unknown regulatory functions of EVs associated with the microenvironment of NSCs or proliferating astrocytes by assessing the behaviours of the visualized MVBs and EVs.

### EVs from eNSCs transfer to eNSCs and astrocytes *in vitro*

**Fig. 6.**
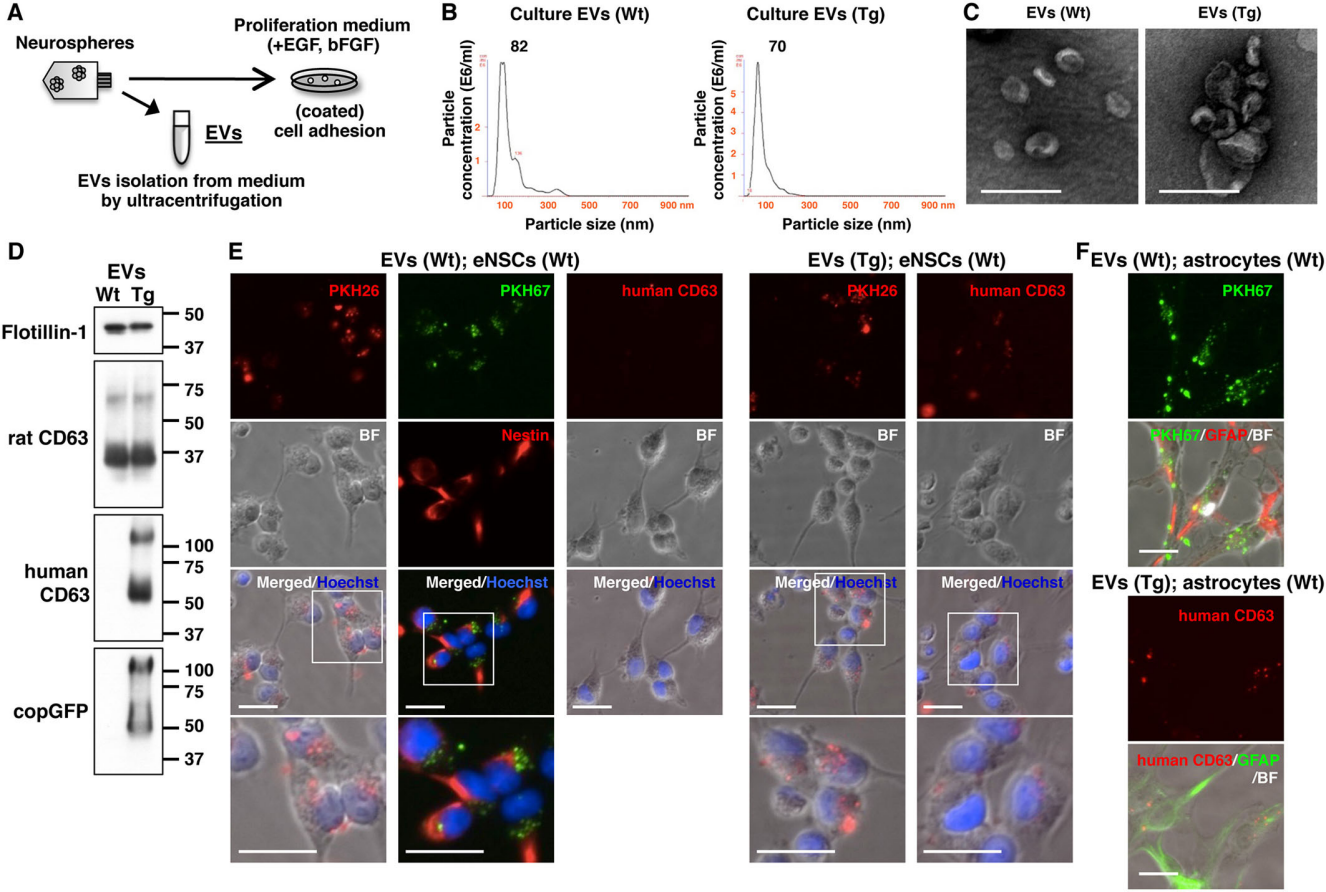
**Transfer of eNSC-derived EVs into recipient cells *in vitro*.** (A) Schematic representation of the protocol for collection of EVs from the conditioned medium of eNSCs. The eNSCs as recipient cells were cultured on pre-coated dishes in proliferation medium. (B) Size distribution of the eNSC-derived EVs obtained from the conditioned medium of Wt and Tg rat cells by the NanoSight system. The experiments were performed four times. (C) Electron microscopic images of the EVs. Scale bars: 200 nm. The experiment was performed once. (D) Western blot analysis showed the EV markers flotillin-1 and endogenous rat CD63 expression in the isolated EVs in addition to exogenous human CD63-GFP in the Tg EVs. The experiment was performed once. (E) The Wt EVs were pre-labelled with PKH26 (red) or PKH67 (green), and Tg EVs were labelled with PKH26. Wt eNSCs were incubated with the EVs for 8 h. Furthermore, the EVs from Tg cells were detected using anti-human CD63 (red) after having been transferred into eNSCs. The cells were immunostained with antibodies against nestin (red), showing PKH67 signals around nuclei (blue). The experiments were performed twice. (F) The Wt and Tg EVs were incubated with primary astrocytes of the cerebral cortex of Wt rats. Wt EVs were labelled with PKH67, whereas Tg EVs were detected by immunostaining of human CD63 (red). Astrocytes were recognized by the antibody against GFAP. The experiment was performed once. Scale bars: 20 µm.

We next analysed the transmission of EVs from eNSCs to recipient cells *in vitro*. EVs were prepared from the conditioned media of neurospheres cultured for 4 days (Fig. 6A). Isolated EV pellets showed a size distribution with peaks between 50 and 100 nm (Fig. 6B). The particles that were imaged by electron microscopy (EM) were observed to be mainly between 50 and 150 nm in size and showed typical cup-shaped structures (Fig. 6C). The EV markers rat CD63 and flotillin-1 were detected by western blotting in both WT and Tg eNSC-derived EVs, whereas human CD63 and copGFP were expressed only in Tg eNSC-derived EVs (Fig. 6D).

**Fig. 7.**
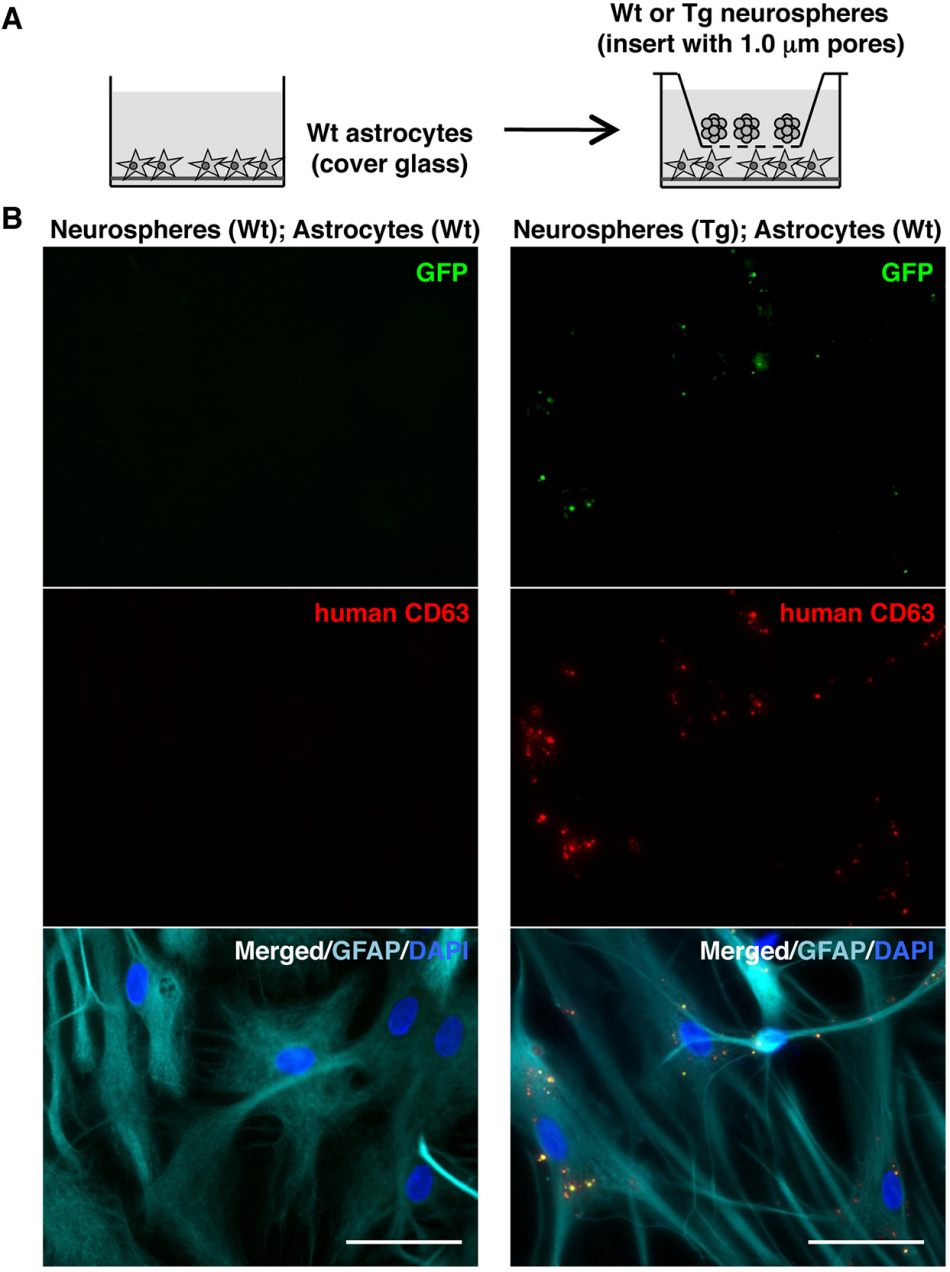
***In vitro* transfer of neurosphere-derived EVs to astrocytes in the co-culture system.** (A) A schematic illustration of the co-culture protocol. Wt astrocytes as recipient cells were cultured on pre-coated coverglass on the bottom of wells, and then they were cultured for 4 days with Tg or Wt neurospheres put into the culture insert with 1.0 µm pores. (B) EVs derived from Tg neurospheres in the Wt astrocytes were detected by GFP (green) and immunoreactivity of human CD63 (red). Astrocytes were stained with an antibody against GFAP (cyan). Nuclei were visualized by DAPI (blue). All the experiments were duplicated in different plates using sister cultures. Scale bars: 50 µm.

We next examined whether the collected EVs could be incorporated into eNSCs and astrocytes. The eNSCs as recipient cells were cultured in proliferation medium on pre-coated 24-well plates until cells adhered on the bottom (Fig. 6A). The collected EVs were incubated with eNSCs *in vitro* for 8 h (Fig. 6E). Wt EVs were pre-labelled with the fluorescent lipid dye PKH26 (red) or PKH67 (green). We observed fluorescent signals in the cells, and PKH67-labelled Wt EVs showed highly intense fluorescent signals around nuclei in nestin-positive cells (Fig. 6E). When Tg EVs were stained with PKH26, the internalization of PKH26-labelled Tg EVs also showed highly intense fluorescent signals around nuclei (Fig. 6E). Human CD63 immunofluorescence was clearly detected in the cells incubated with Tg EVs but not in those incubated with Wt EVs (Fig. 6E). We next determined the incorporation of EVs into different cell types. We prepared astrocytes from the cerebral cortex of new-born rats at P1. Wt and Tg EVs were taken up by GFAP-positive cells after 11 h (Fig. 6F). To confirm the transfer of EVs between cells in more physiological conditions, we performed co-culture of Tg neurospheres and Wt astrocytes, using cell culture inserts with 1.0 µm pores for 4 days (Fig. 7A). Both GFP signals and human CD63 immunoreactivity were observed in Wt astrocytes co-cultured with Tg neurospheres, whereas they were not detected in the astrocytes cultured with Wt neurospheres (Fig. 7B).

Thus far, studies of EVs in the CNS have demonstrated neuron-neuron, glia-glia and neuron-glia intercellular communication via EVs. It has been reported that neuronal EVs were transferred into astrocytes and that neuronal EV-derived miRNA-124a, the least expressed miRNA in astrocytes, could regulate the expression of the astroglial glutamate transporter GLT1 (Morel et al., 2013). By contrast, oligodendroglial EVs were mainly taken up into neurons and microglia (Fitzner et al., 2011; Frühbeis et al., 2013). However, the functions of NSC-derived EVs, including eNSCs, are poorly understood. In the present study, we found that EVs released from eNSCs were transferred into astrocytes. NSC-derived EVs might be involved in the maintenance of self-renewal of NSCs because their regeneration ability by the transfer of stem cell-derived mRNA was reported in a study of other stem cell-derived EVs (Katsman et al., 2012); EVs from a human NSC line shuttled miRNA-1246, which is known to play a role in regulating cell growth (Stevanato et al., 2016). It is also possible that NSC-derived EVs activate some signalling pathways associated with neurogenesis in astrocytes, because NSCs and astrocytes in the adult hippocampus have a close relationship to promote neurogenesis. The Wnt3a protein released from astrocytes stimulates target genes in NSCs through Wnt signalling, and regulates neuronal differentiation (Kuwabara et al., 2009). Although more work is required to examine whether EVs released from the NSCs of adult brains are also taken up into astrocytes, future experiments will elucidate the function of NSC-derived EVs in astrocytes by miRNA analysis to find the most abundant miRNAs in the EVs and their potential targets in astrocytes.

Interestingly, our Tg rats showed GFP expression in the hair follicles after birth. Li et al. (2003) reported that exogenous GFP expression driven by the promoter for nestin, which is a marker for neural progenitor cells, was observed in stem cells of hair follicles. In nestin-driven GFP (ND-GFP) mice, it was also revealed that ND-GFP-expressing cells in the whisker follicle have a role in the growth of the follicle sensory nerve (Mii et al., 2013). In addition, nestin-expressing cells in the hair follicle could potentially differentiate into neurons and cardiac muscle cells (Amoh et al., 2005; Yashiro et al., 2015).

EVs have become the focus of extensive investigation as important factors in the communication between transplanted stem cells and host tissues in stem cell therapies (Barile et al., 2014; Katsuda et al., 2013). Cossetti et al. (2014) reported that EVs derived from NSCs exposed to inflammatory cytokines carried interferon-γ (IFN-γ) bound to IFN-γ receptor 1 on their surface, and then activated the STAT1 signalling pathway in target cells *in vitro*, implying functional communication between transplanted NSCs and the host immune system via EVs. Our animal model might potentially provide a visual approach to enable the study of EV-dependent communication in the transplanted tissues in stem cell therapies.

We demonstrated here that GFP-labelled EVs released by NSCs were incorporated into astrocytes *in vitro*, although further studies regarding the *in vivo* transfer of EVs in the Sox2/human CD63-GFP Tg rats are needed. Recent technological advances in *in vivo* imaging have made it possible to monitor the real-time release of EVs from transplanted human tumour cells in the mammary glands of mice (Zomer et al., 2015). With such methods, our Tg rats with GFP labels on EVs will provide significant advantages for studying the molecular processes involved in the release of EVs from NSCs and their reception by other cell types both *in vitro* and *in vivo*, especially in the developing brain.

## MATERIALS AND METHODS

### Animals

All animal experiments were conducted in accordance with the institutional guidelines of the Animal Ethics Committee for the care and use of animals, and all experiments were carried out in accordance with the approved guidelines of the National Institute of Neuroscience, National Center of Neurology and Psychiatry and National Cancer Center Research Institute, Japan.

### Production of Sox2/human CD63-GFP Tg rats

Wistar rESCs were used to generate Tg rats. The rESCs were derived from cell lines established by Kawamata and Ochiya (2010). The rESCs were maintained on mitomycin-C-treated neomycin-resistant mouse embryonic fibroblasts (MEFs) (Millipore, MA, USA) in DMEM medium (containing high-glucose, GlutaMAX and pyruvate) (Life Technologies, CA, USA), supplemented with 20% FBS (ES Cell Qualified Fetal Bovine Serum; Life Technologies), 0.1 mM 2-mercaptoethanol (Sigma-Aldrich, MO, USA), 1% MEM non-essential amino acids solution (Life Technologies), 1% antibiotic-antimycotic solution (Life Technologies) and the following four inhibitors: 10 μM Y-27632 (Wako,Tokyo, Japan), 1 μM PD0325901 (Axon Medchem, Groningen, The Netherlands), 0.5 μM A-83-01 (Tocris, Bristol, UK) and 3 μM CHIR99021 (Axon Medchem; YPAC medium), as described previously.

The *Sox2* promoter fragment used in the present study consists of the rat *Sox2* 5′ flanking sequences (6647 bp) obtained from the genomic DNA of Wistar rats by PCR (forward primer, 5′-AGCGGCCTCC- TGATGTCAACAGA-3′; reverse primer, 5′-TATTCTCCGCCAGATCTCCGCGC-3′) using KOD Plus Neo (Toyobo, Osaka, Japan). The 6.65 kb *Sox2* 5′ flanking sequence includes 5.6 kb *Bgl*II fragment, which is a region homologous with the mouse *Sox2* promoter (D'Amour and Gage, 2003). A DNA fragment encoding human CD63-copGFP (human CD63-GFP) from a pCT-CMV-CD63-GFP vector (System Biosciences, CA, USA) and the *Sox2* promoter were subcloned into a pECFP-1 plasmid (Clontech, Shiga, Japan). The Sox2/human CD63-GFP plasmid was linearized by digestion with *Sal*I and was then transfected into Wistar rESCs using a Mouse ES Cell Nucleofector Kit (Lonza, Basel, Switzerland) and an Amaxa Nucleofector as described previously (Kawamata and Ochiya, 2010). The rESCs were seeded onto mitomycin-C-treated neomycin-resistant MEFs in YPAC medium with 2% Matrigel (Becton Dickinson, Franklin Lakes, NJ, USA) at 37°C in air enriched with 5% CO_2_, and 1 day later, 0.2 mg/ml G418 (Sigma-Aldrich) was added to the culture medium for the selection of transfected cells. The rESC colonies showing GFP fluorescence were selected and amplified.

Rat embryos were prepared using the protocol described previously (Kawamata and Ochiya, 2010). Blastocysts were collected from LEA×LEA or Wistar×LEA matings 4.5 days post-coitus (dpc). Approximately 12 rESCs were injected into each blastocyst. The injected blastocysts were surgically transferred into the uterine horns of pseudopregnant Wistar females at 3.5 dpc. Chimaeric rats were confirmed by coat colour chimaerism. The potential of rESCs for germline transmission was examined by the coat colour of F1 rats resulting from mating with Wistar rats, and the inheritance in the offspring was assessed by GFP fluorescence and PCR analysis of genomic DNA isolated from ear snips.

### Primary eNSC culture and differentiation

All eNSCs were obtained from telencephalon tissues of E14 Wistar Wt and Tg [Wistar-esTgN(Sox2/CD63-GFP)3NCCRI] rats. Isolated eNSCs were cultured on noncoated tissue culture plastic as neurospheres in KBM neural stem cell proliferation medium (Kohjin Bio, Saitama, Japan) with 0.2% KBM supplement containing EGF and bFGF (Kohjin Bio) at 37°C in air enriched with 5% CO_2_, as described previously (Yoshimura et al., 2016a). After 3 or 4 days, neurospheres were dissociated using trypsin (Sigma-Aldrich) and passaged. Differentiation was carried out by seeding on tissue culture dishes pre-coated with 0.2% polyethyleneimine (PEI) (Wako). Differentiation medium consisted of KBM containing 2% B-27 serum-free supplement containing vitamin A (Life Technologies) without EGF and bFGF. Dissociated cells were plated at a density of 1.8×10^6^ cells per 35 mm culture dish (Becton Dickinson). For immunocytochemistry, 1.4×10^5^ cells/0.2 ml were reseeded on pre-coated glass-bottomed dishes (Matsunami, Osaka, Japan). The culture medium was changed 3 or 4 days after plating, and differentiation proceeded for 7 days. To promote the proliferation of differentiated astroglial cells, culture medium was replaced with astroglial culture medium (described in the next section) at 6 days after differentiation.

### Primary astrocyte culture

Primary astrocytes were prepared from the cerebral cortex of Wistar rats on postnatal day 1 (P1) as described previously (Numakawa et al., 2011). Dissociated astroglial cells were plated onto noncoated tissue culture flasks and cultured in the presence of 0.1 µg/ml mouse recombinant EGF (PeproTech, NJ, USA) in an MEM (Life Technologies)-based growth medium containing 5% FBS (Biological Industries, Beit Haemek, Israel), 20 mM glucose, 25 mM NaHCO_3_ and 0.5 mM glutamine at 37°C in air enriched with 5% CO_2_. Cells were maintained in the astroglial culture medium until EV transfer analysis, and the culture medium was changed once a week.

### Immunoblotting

The composition of the protein lysis buffer was as follows: 1% SDS, 10 mM Tris-HCl (pH 7.5), 5 mM EDTA, 10 mM sodium pyrophosphate, 10 mM NaF, 1 mM phenylmethylsulfonyl fluoride and 2 mM Na_3_VO_4_. Samples of tissues and cells were sonicated on ice, and then lysates were cleared by centrifugation. The lysates were dissolved in SDS sample buffer without 2-mercaptoethanol (Wako) after determination of the protein concentration using a Pierce BCA Protein Assay Kit (Life Technologies).

After being blocked with 5% skimmed milk, membranes were probed with specific primary antibodies to CNPase (1:1000; ab6319; Abcam, Cambridge, UK), copGFP (1:10,000; AB501; Evrogen, Moscow, Russia), flotillin-1 (1:500; 610820; Becton Dickinson), GFAP (1:1000; AB5804; Millipore), GluR1 (1:1000; AB1504; Millipore), human CD63 (1:250; 556019; Becton Dickinson), rat CD63 (1:250; MCA4754GA; AbD Serotec, San Jose, CA, USA), SOX2 (1:1000; ab97959; Abcam), syntaxin 1 (1:1000; S0664; Sigma-Aldrich) and TUJ1 (1:1000; MMS-435P; Berkeley Antibody Company, Berkeley, CA, USA) followed by incubation with peroxidase-conjugated anti-mouse IgG (1:1000; Jackson ImmunoResearch, West Grove, PA, USA) or rabbit IgG (1:1000; Rockland, Limerick, PA, USA) secondary antibody. β-actin (1:5000; A5441; Sigma-Aldrich) was used as a loading control. Signals were detected using chemiluminescent reagents (ImmunoStar; Wako).

### Immunocytochemistry

Cultured cells were fixed with 4% paraformaldehyde (PFA) in PBS for 15 min following a pretreatment with 2% PFA in the culture medium for 30 min at room temperature. After being washed, cells were incubated in PBS containing 0.2% Triton X-100 (Sigma-Aldrich) and 10% FBS with primary antibodies at 4°C overnight. Primary antibodies specific to the following proteins were used: CNPase (mouse IgG_1_ 1:250; ab6319; Abcam), GFAP (rabbit IgG 1:1000; ab7260; Abcam), human CD63 (mouse IgG_1_ 1:200; 556019; Becton Dickinson), MAP2 (mouse IgG_1_ 1:200; M1406; Sigma-Aldrich), nestin (mouse IgG_2a_, 1:200; MAB2736; R&D Systems, MN, USA), rat CD63 (mouse IgG_1_ 1:200; MCA4754GA; AbD Serotec) and SOX2 (rabbit IgG 1:200; ab97959; Abcam). After cells were stained with Alexa Fluor 546 mouse IgG_1_ (1:2000; Life Technologies), Alexa Fluor 546 mouse IgG_2a_ (1:2000; Life Technologies), Alexa Fluor 546 rabbit IgG (1:2000; Life Technologies), Alexa Fluor 488 mouse IgG_1_ (1:200; Life Technologies) and Alexa Fluor 488 rabbit IgG (1:200; Life Technologies), they were observed through a BIOREVO BZ-9000 (Keyence, Osaka, Japan) fluorescence microscope. Hoechst 33342 was used to stain cell nuclei.

### Immunohistochemistry

Head samples were collected from Wt and Tg rats at E16, and they were fixed with 4% PFA overnight at 4°C. They were washed in PBS, and then cryoprotected in 30% sucrose. After freezing in OCT compound, the frozen brains were sectioned using a cryostat into 10 µm slices. Primary antibody for SOX2 (1:200; GTX101507; GeneTex, LA, USA) was diluted in blocking solution (0.25% Triton and 2% BSA in PBS) or (0.05% saponin and 2% BSA in PBS). Note that use of saponin gave better results to minimize quenching of GFP signals. All images were captured using an FV1000 confocal microscope (Olympus, Tokyo, Japan).

### Characterization of EVs isolated from serum and eNSC culture medium

Blood samples were collected from Wt and two Tg adult female rats [Wistar-esTgN(Sox2/CD63-GFP)3NCCRI and Wistar-esTgN(CAG/CD63-GFP)3NCCRI] (Yoshimura et al., 2016b). Blood was centrifuged at 2000 ***g*** for 10 min. The collected serum was further centrifuged twice at 10,000 ***g*** for 15 min to remove blood cells and debris. The resultant supernatant was transferred to ultracentrifuge tubes, and then EVs were isolated by ultracentrifugation at 210,000 ***g*** for 70 min at 4°C using a Beckman SW41Ti rotor (Beckman, Fullerton, CA, USA). The pellets were washed with PBS and ultracentrifuged again.

Culture media were sampled from the eNSC culture prepared from Wt and Tg [Wistar-esTgN(Sox2/CD63-GFP)3NCCRI] rats. Rat eNSCs, which were passaged once, were cultured in proliferation medium for 4 days on noncoated tissue culture flasks. To isolate EVs, culture supernatant was centrifuged at 2000 ***g*** for 10 min and then filtered through a 0.22 µm membrane filter (Millipore) to remove cellular debris. EVs were collected by ultracentrifugation at 210,000 ***g*** for 70 min at 4°C and washed with PBS. The collected EV samples were diluted 100-fold with PBS for the analysis. Analysis of the size distribution of EVs was carried out with the Nanosight LM10HS (NanoSight, Amesbury, UK), as described previously (Yoshioka et al., 2013). For EM observation, eNSC-derived EVs were plated on collodion-carbon-coated grids and negatively stained with uranyl acetate. The images were captured using a transmission EM (Tecnai Spirit; FEI, OR, USA).

### Analysis of EV transfer *in vitro*

Isolated EVs from eNSC cultures of Wt or Tg [Wistar-esTgN(Sox2/CD63-GFP)3NCCRI] rats were visualized using a PKH26 or PKH67 fluorescence labelling kit (Sigma-Aldrich). Rat eNSCs were reseeded on coated 24-well glass-bottomed plates in proliferation medium, and astrocytes prepared from rat cerebral cortex were reseeded on noncoated 24-well glass-bottomed plates in astroglial culture medium. The EVs were incubated with the eNSCs or astrocytes on the 24-well plates at 37°C in air enriched with 5% CO_2_. On washing with PBS, the cultured cells were fixed with 4% PFA. Anti-human CD63 antibody was used to detect the EVs from Tg eNSCs in the recipient cells. Internalization of EVs was captured by using a Biorevo BZ-9000 fluorescence microscope.

In the co-culture system, Wt astrocytes were plated onto a micro-coverglass (Matsunami) pre-coated with PEI, and they were incubated in the bottom of 24-well plates. A cell culture insert with 1.0 µm pores (Falcon; Corning, NY, USA) was set on each well. Tg or Wt neurospheres were put into the insert and cultured in KBM medium with 0.2% KBM supplement at 37°C in air enriched with 5% CO_2_. After 4 days of co-culture, the Wt astrocytes were fixed with 4% PFA for 20 min. For immunocytochemistry, anti-human CD63 and GFAP antibodies were used. TrueBlack Lipofuscin Autofluorecence Quencher (Biotium, CA, USA) was used after immunostaining to quench lipofuscin autofluorescence in astrocytes. The images were captured using an Axiovert 200 fluorescence microscope (Carl Zeiss, Oberkochen, Germany).

## Supplementary Material

Supplementary information
