# Supplementary material for: The Sox2 promoter-driven CD63-GFP transgenic rat model allows tracking of neural stem cell-derived extracellular vesicles
Source: Dis Model Mech. 2018 Jan 1;11(1):dmm028779. doi: 10.1242/dmm.028779 (PMC5818070; doi:10.1242/dmm.028779)
Supplement: Supplementary information [file dmm-11-028779-s1.pdf]

Fig. S1.

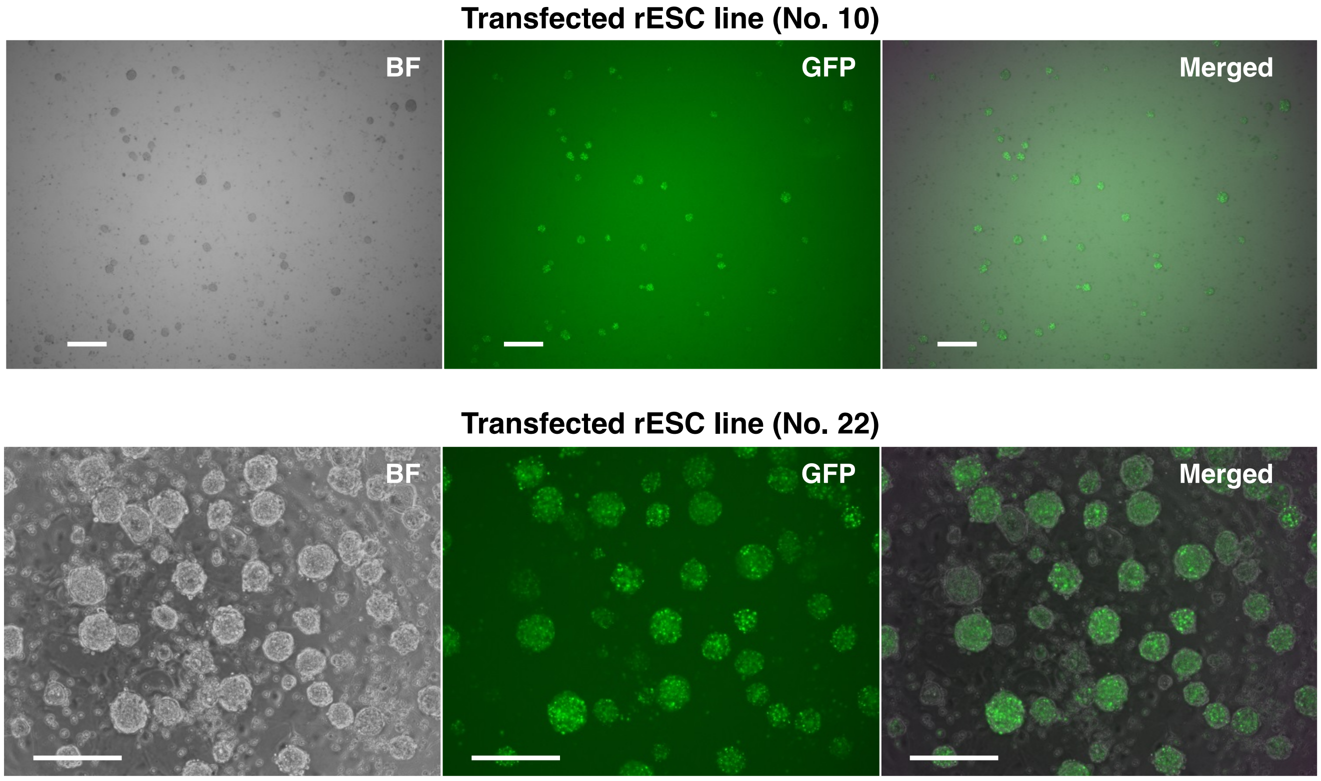

Fig. S1. Transfection of Sox2/human CD63-GFP gene into rESCs. Photomicrographs of two transfected rESC lines (No. 10 and No. 22) with ubiquitous expression of GFP. Scale bar: 300 μm.

Disease Models & Mechanisms • Supplementary information

Fig. S2.

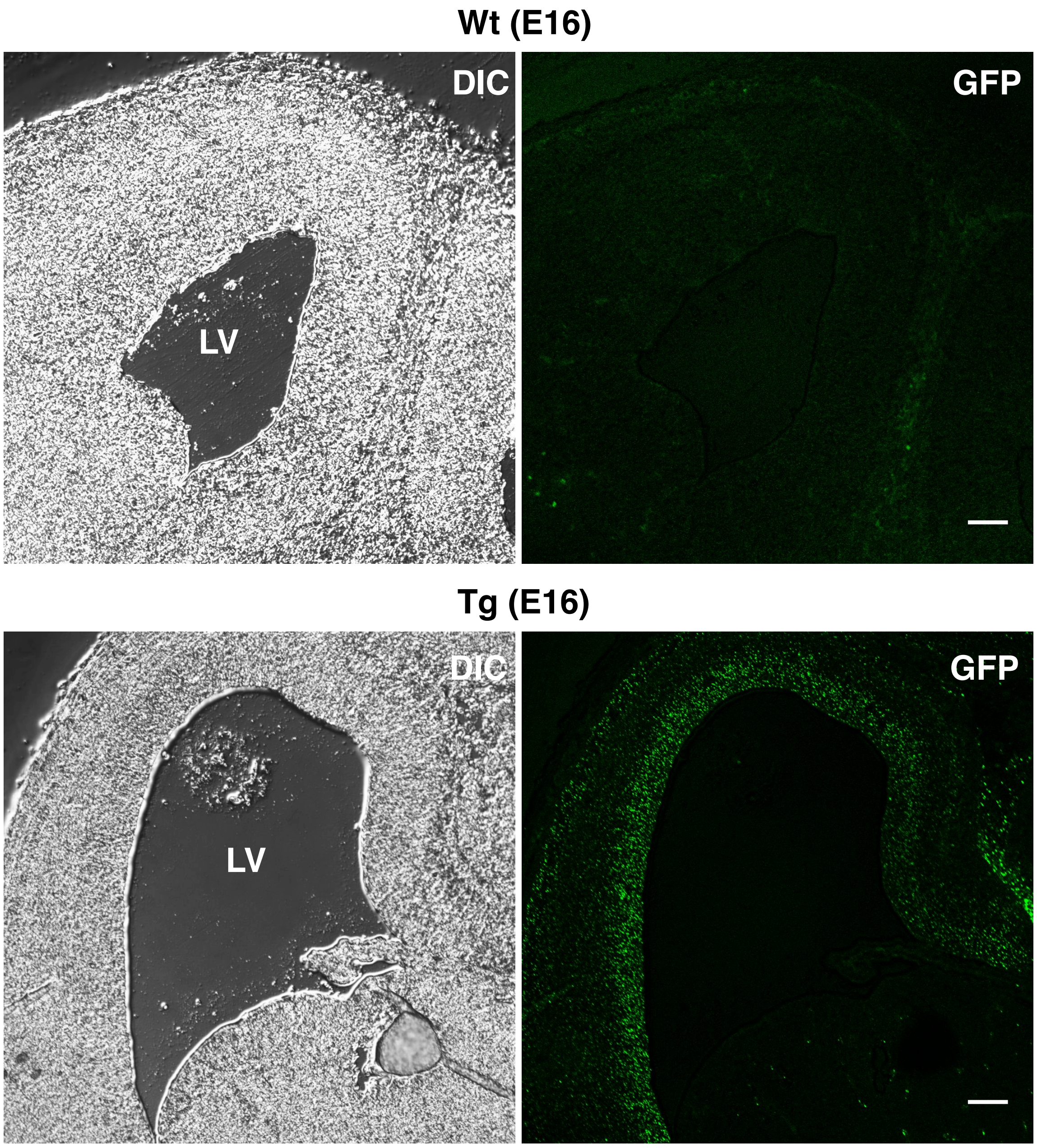

Fig. S2. GFP expression in the telencephalon of Tg rat embryo. Coronal sections of the Wt and Tg telencephalon. GFP signals were clearly observed around the lateral ventricle (LV) of the telencephalon in Tg rat. Scale bar: 100 μm.

Fig. S3.

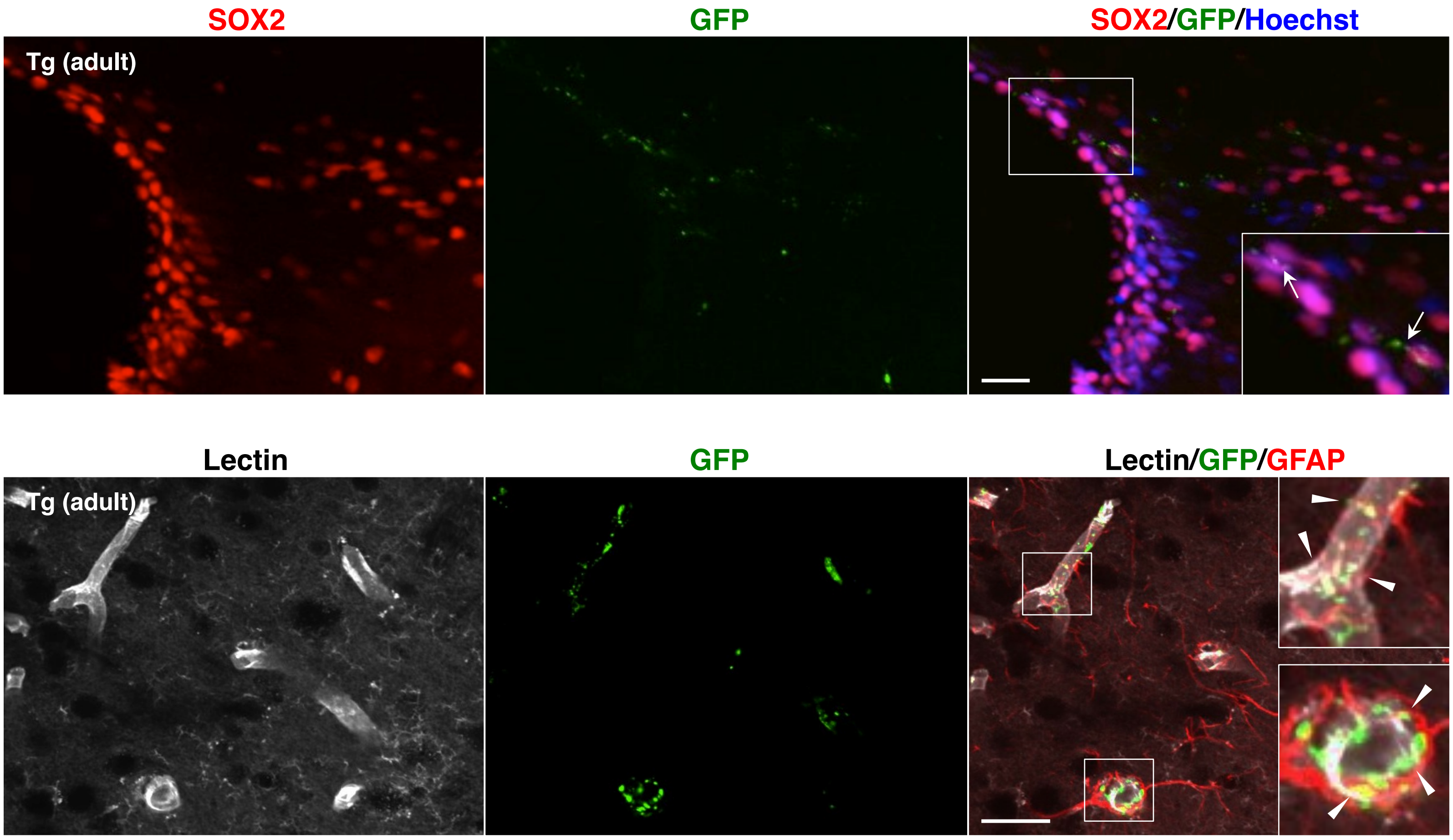

Fig. S3. GFP expression in the adult Tg rat cortex. About 7 month-old Wt and Tg female rats were perfused with PBS and 4% PFA, and the removed brains were postfixed in 4% PFA overnight at 4°C. The fixed brains were sectioned using a microslicer into 50 µm slices. Neural stem cells and astrocytes were identified by immunostaining with antibodies against SOX2 (GeneTex) and GFAP (G3893, Sigma-Aldrich), respectively. The blood vessels were stained with lectin antibody (B-1175, Vector Laboratories, CA, USA). Nuclei were stained with Hoechst 33342 (blue). Scale bar: 25 µm.

Disease Models & Mechanisms • Supplementary information

Fig. S4.

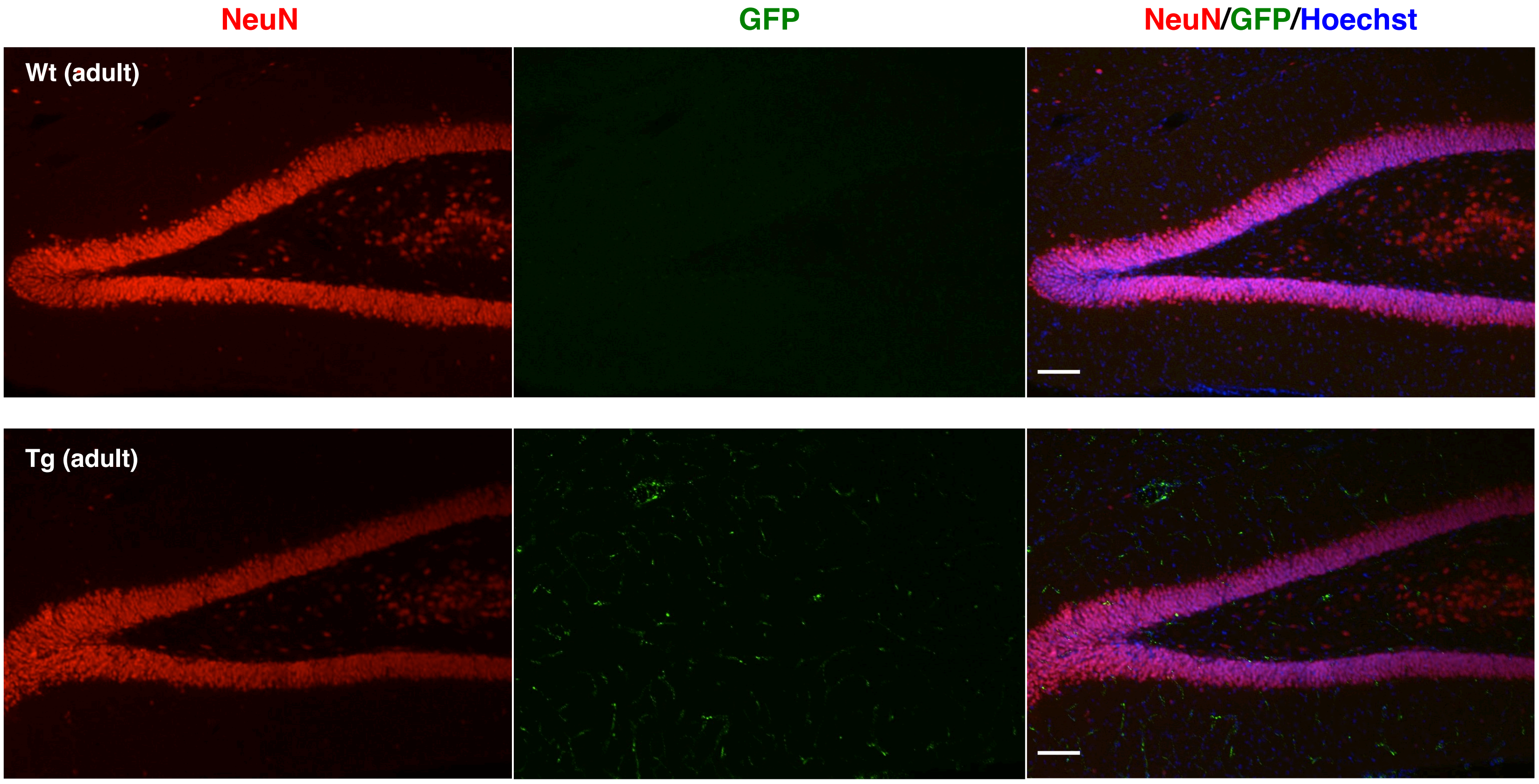

Fig. S4. Distribution of NeuN-positive neurons in the hippocampus of adult Wt and Tg rats. The sagittal sections of the hippocampus from 7 month-old Wt and Tg female rats. Neural cells were identified by an antibody against NeuN (ab177487, Abcam). Nuclei were stained with Hoechst 33342 (blue). Scale bar: 100 μm.

Fig. S5.

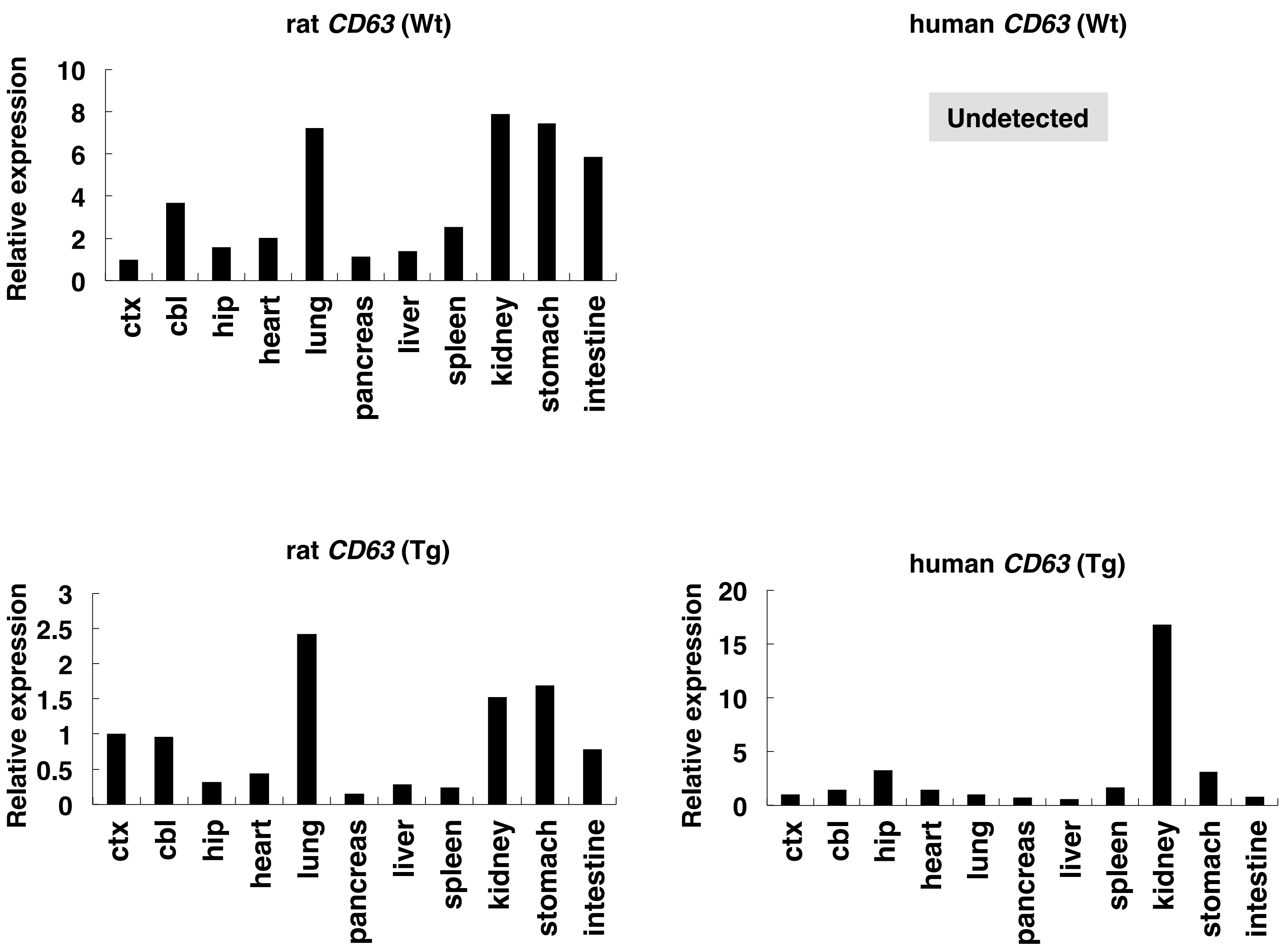

Fig. S5. Quantitative PCR analysis of rat *CD63* and human *CD63* mRNA expression levels in the major organs of new-born Wt and Wistar-esTgN(Sox2/CD63-GFP)3NCCRI Tg rats. The RNA samples were purified from a Wt (postnatal day 1) and a Tg (postnatal day 2) rats by RNeasy Mini kit (QIAGEN, Venlo, Netherlands). Complementary DNAs (cDNAs) for mRNAs were generated using SuperScript VILO cDNA synthesis kit (Life Technologies). The cDNAs were subjected to StepOnePlus Real Time PCR System (Applied Biosystems, MA, USA) with TaqMan Gene Expression Assays; rat *CD63* (ID: Rn01529322) and human *CD63* (ID: Hs01041238). The relative expression levels of mRNAs were normalized to *gapdh* (ID: Rn01775763) mRNA levels and calculated by the $\Delta\Delta C_t$ method. A high level of human *CD63* was detected in the hippocampus, stomach and especially kidney of the Tg rats but not in any tissues of Wt rats. Abbreviations: ctx, cortex; cbl, cerebellum; hip, hippocampus.

Fig. S6.

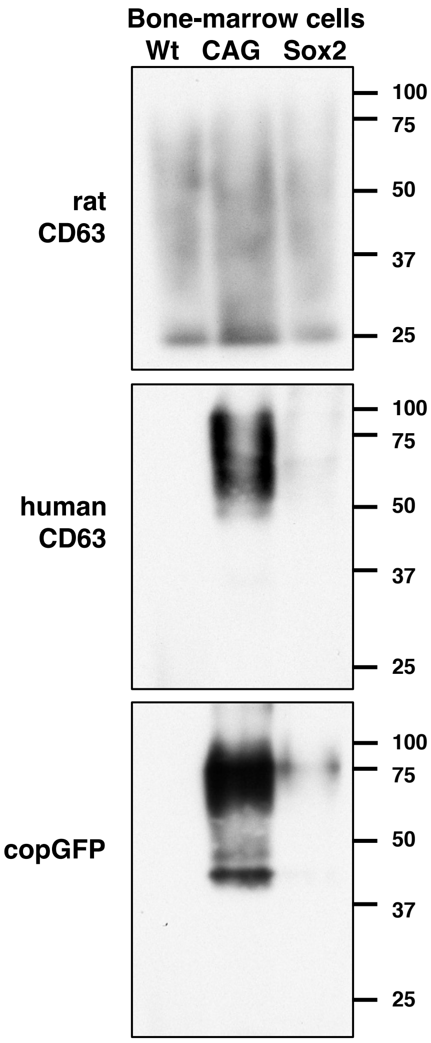

Fig. S6. Human CD63-GFP expression in the bone-marrow cells isolated from Wt and two Tg rats (Wistar-esTgN(Sox2/CD63-GFP)3NCCRI and Wistar-esTgN(CAG/CD63-GFP)3NCCRI). Bone-marrow cells were obtained from the femurs of adult female rats. Cells were collected in RPMI1640 medium (Life Technologies) and then centrifuged at 1,400 rpm for 5 minutes. The pellets were washed with the medium and PBS. The cell lysates were analysed by Western blot analysis for endogenous rat CD63, exogenous human CD63 and copGFP. The signals of human CD63 and copGFP were barely detected in the bone-marrow cells from Sox2/CD63-GFP rats, whereas these signals were strongly detected in that of CAG/CD63-GFP rats.

Disease Models & Mechanisms • Supplementary information

Fig. S7.

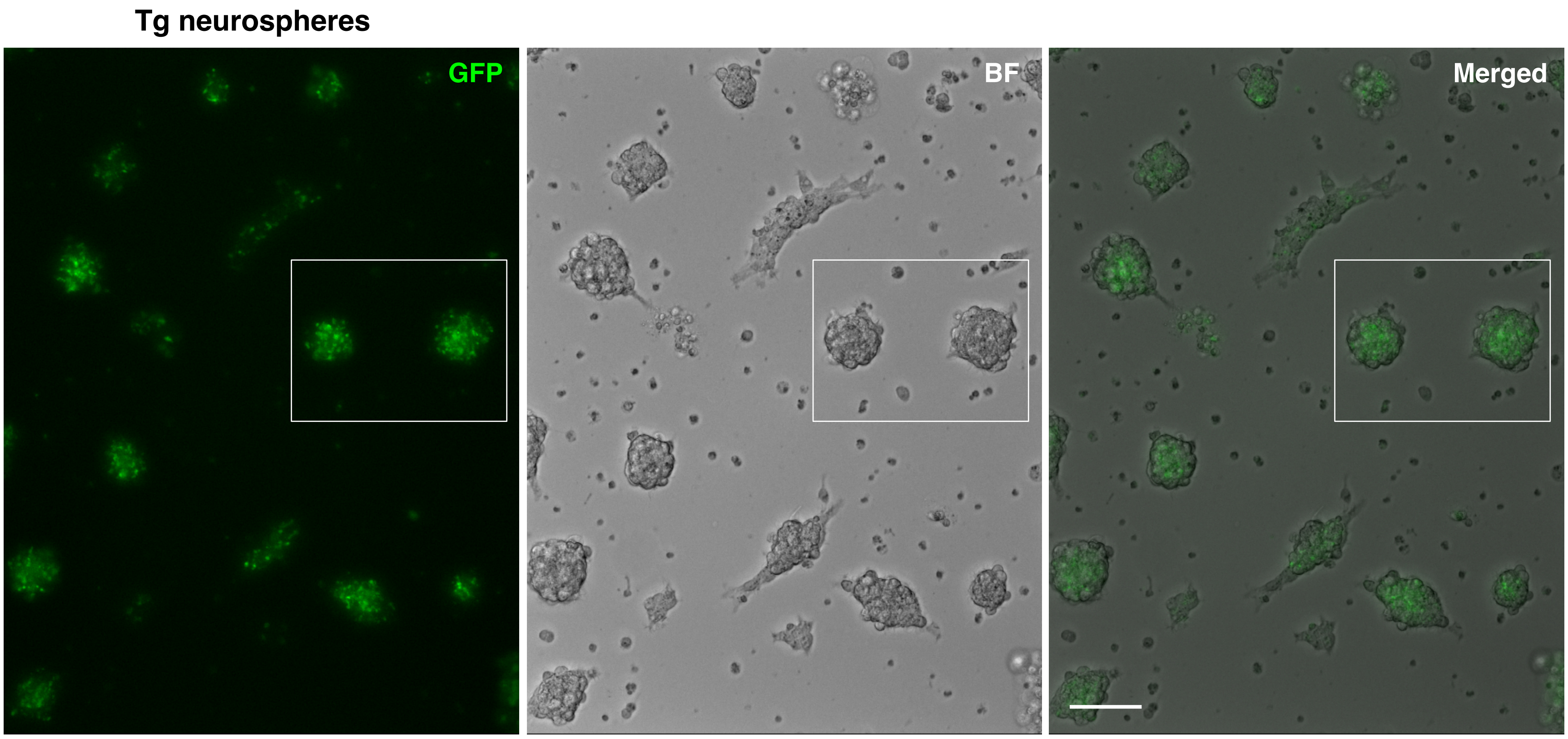

Fig. S7. GFP-positive neurospheres obtained from Tg rats at E14. The wide-field images of Fig. 5B. The neurospheres shown in Fig. 5B are indicated by white square. Scale bar: 50 μm.

Fig. S8.

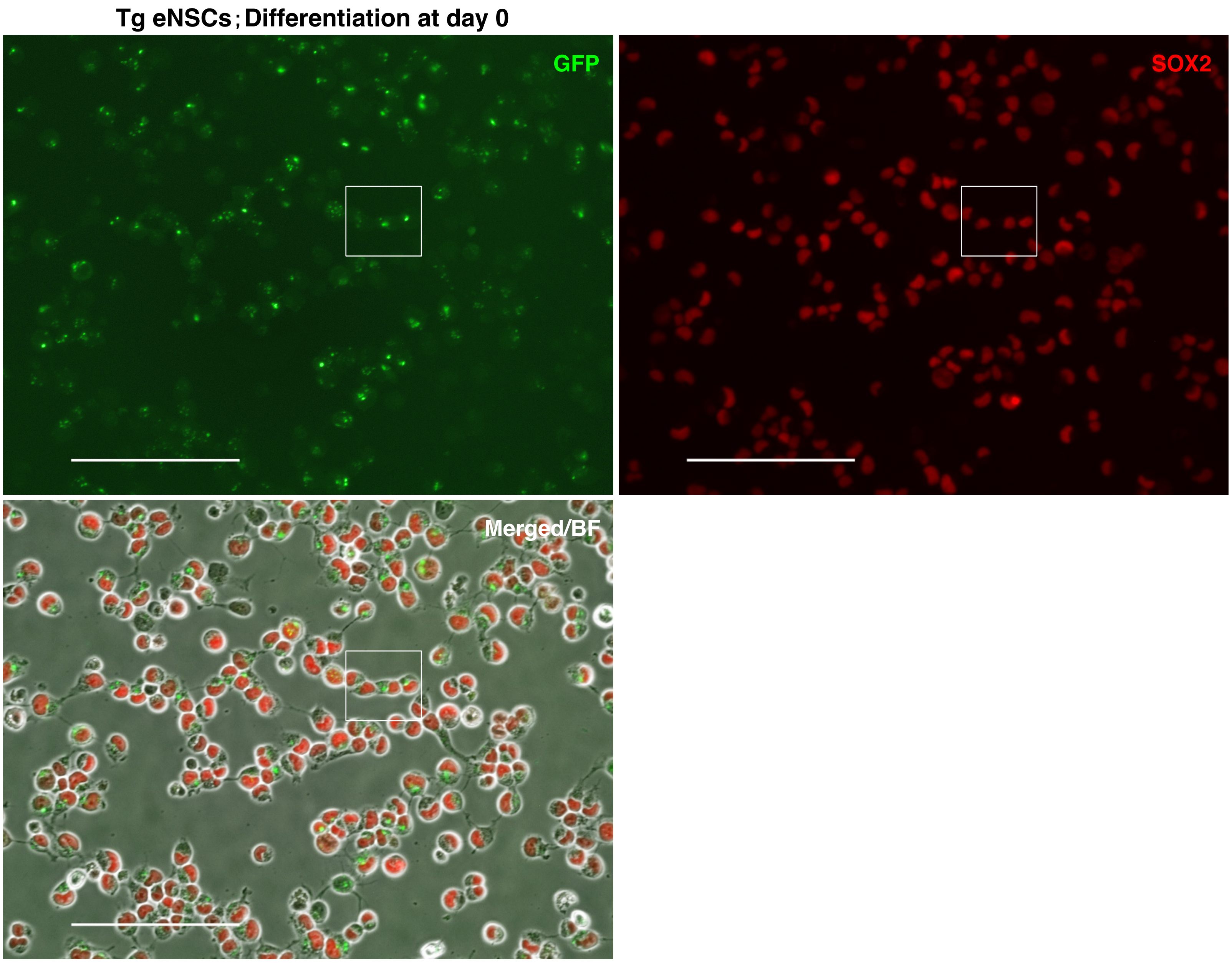

Fig. S8. GFP expression in cultured Tg eNSCs at day 0. The wide-field images of Fig. 5D. GFP signals were detected in the SOX2-positive cells. The images shown in Fig. 5D are indicated by white square. Scale bar: 100 μm.

Disease Models & Mechanisms • Supplementary information

Fig. S9.

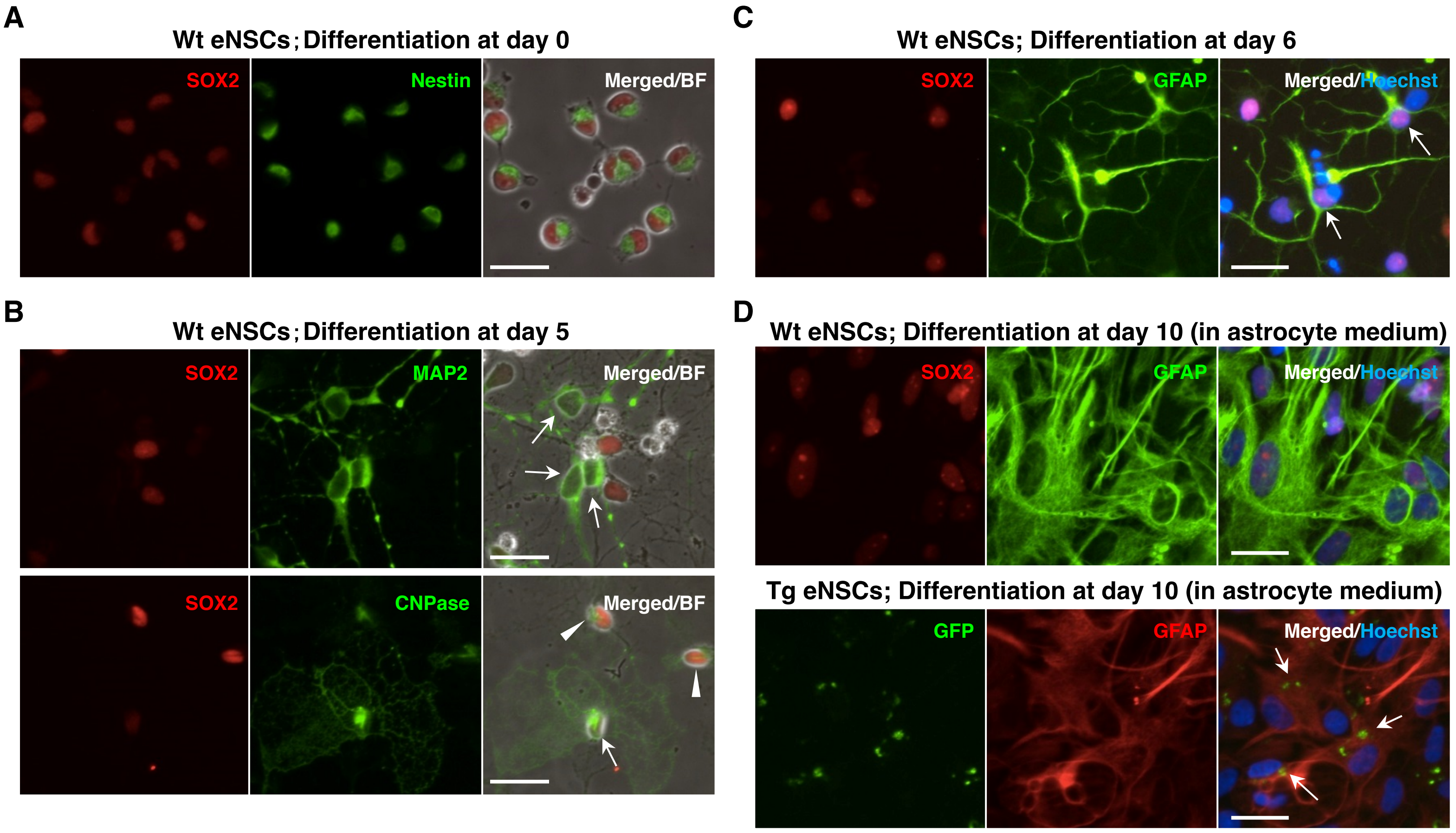

Fig. S9. Differentiated astrocytes maintained SOX2 expression and GFP expression was resumed as astrocytes proliferated. (A-D) Immunostaining of the rat eNSCs during differentiation with antibodies against SOX2, nestin, MAP2, CNPase and GFAP. (A) Nestin-positive cells showed SOX2 expression. (B) MAP2-positive and CNPase-positive cells showed reduced SOX2 expression (arrows). SOX2 expression was maintained in the cells expressing immature CNPase signals (arrowheads). (C) In contrast, GFAP-positive cells showed SOX2-positive nuclei (arrows). (D) After the culture medium was replaced with astrocyte medium on day 6, GFAP-positive cells dramatically proliferated and GFP fluorescence was detected in the GFAP-positive cells of Tg rats (arrows). Nuclei were stained with Hoechst 33342 (blue). Scale bar: 25 µm.

Disease Models & Mechanisms • Supplementary information
